# Alteration of the intestinal microbiome characterizes preclinical inflammatory arthritis in mice and its modulation attenuates established arthritis

**DOI:** 10.1038/s41598-017-15802-x

**Published:** 2017-11-15

**Authors:** Rebecca Rogier, Heather Evans-Marin, Julia Manasson, Peter M. van der Kraan, Birgitte Walgreen, Monique M. Helsen, Liduine A. van den Bersselaar, Fons A. van de Loo, Peter L. van Lent, Steven B. Abramson, Wim B. van den Berg, Marije I. Koenders, Jose U. Scher, Shahla Abdollahi-Roodsaz

**Affiliations:** 1 Department of Rheumatology, Radboud University Medical Center, Nijmegen, The Netherlands; 2 Department of Medicine, Division of Rheumatology, New York University School of Medicine, New York, United States

## Abstract

Perturbations of the intestinal microbiome have been observed in patients with new-onset and chronic autoimmune inflammatory arthritis. However, it is currently unknown whether these alterations precede the development of arthritis or are rather a consequence of disease. Modulation of intestinal microbiota by oral antibiotics or germ-free condition can prevent arthritis in mice. Yet, the therapeutic potential of modulation of the microbiota after the onset of arthritis is not well characterized. We here show that the intestinal microbial community undergoes marked changes in the preclinical phase of collagen induced arthritis (CIA). The abundance of the phylum Bacteroidetes, specifically families S24-7 and Bacteroidaceae was reduced, whereas Firmicutes and Proteobacteria, such as Ruminococcaceae, Lachnospiraceae and Desulfovibrinocaceae, were expanded during the immune-priming phase of arthritis. In addition, we found that the abundance of lamina propria Th17, but not Th1, cells is highly correlated with the severity of arthritis. Elimination of the intestinal microbiota during established arthritis specifically reduced intestinal Th17 cells and attenuated arthritis. These effects were associated with reduced serum amyloid A expression in ileum and synovial tissue. Our observations suggest that intestinal microbiota perturbations precede arthritis, and that modulation of the intestinal microbiota after the onset of arthritis may offer therapeutic opportunities.

## Introduction

Commensal intestinal microbiota have been implicated in several autoimmune diseases including psoriasis, psoriatic arthritis (PsA) and rheumatoid arthritis (RA)^1,2^. PsA and RA are systemic autoimmune diseases characterized by chronic joint inflammation and progressive damage to bone and cartilage. Although their exact etiologies are unknown, both diseases are considered multifactorial and driven by a combination of genetic and environmental factors^3,4^. Several recent studies have shown that the composition of intestinal microbiota is perturbed in patients with recent-onset PsA^5^ and new-onset as well as chronic RA^6–9^. PsA microbiota is characterized by a significant reduction in *Akkermansia, Ruminococcus, and Pseudobutyrivibrio* ^5^. Two studies in patients with new-onset RA have reported significant expansion of *Prevotella copri* prior to immunosuppressive treatment^6,8^. Another study found enrichment of *Lactobacillus salivarius* in RA fecal microbiota, especially during very active disease^7^. This study also found a cluster of metagenomic linkage groups related to *Clostridium asparagiforme*, *Gordonibacter pamelaeae*, *Eggerthella lenta* and Lachnospiraceae to be associated with RA with varying duration (3 months to >10 years)^7^. A fourth study in patients with longstanding, treated RA (mean disease duration 81.6 months) demonstrated increased abundance of *Collinsella*, *Eggerthella*, and *Faecalibacterium* ^9^. Despite this increasing evidence of the altered microbiota in patients with RA and PsA, it is not clear whether the observed perturbations in the intestinal microbiome precede the development of clinical arthritis. Furthermore, the involvement of the microbiome and its targetability during ongoing inflammatory arthritis is less well understood.

One of the most prominent effects of microbiota is to define the balance between the pro-inflammatory CD4^+^ T helper 1 (Th1) and Th17 cells and protective regulatory T (Treg) cells, both at mucosal surfaces and systemically^10,11^. Th17 cells are considered to play a pathogenic role in PsA and RA by producing several proinflammatory cytokines such as interleukin-17 (IL-17), granulocyte-macrophage colony-stimulating factor (GM-CSF) and tumor-necrosis factor (TNFα)^12–16^. Blockade of IL-17A and its receptor is highly efficacious in the treatment of PsA^17,18^ and, despite lack of robust efficacy in RA, can induce sustained American College of Rheumatology 50 (ACR50) response in a subset of patients with RA^19–21^. Th17 cells also induce the production of IL-6, IL-8 and tissue-destructive matrix metalloproteinases by other cells such as macrophages and fibroblasts^13,22–25^. Therefore, modulation of the intestinal microbiome may suppress inflammatory arthritis by reducing Th17 cells and multiple Th17-related related proinflammatory mediators.

Several studies have investigated the role of intestinal microbiota in the onset of inflammatory arthritis in mouse models. Independent lines of evidence have demonstrated that the development of spontaneous arthritis in SKG, K/BxN and IL-1 receptor antagonist deficient (IL-1Ra^−/−^) mice is abrogated under germ-free (GF) conditions^26–28^. Colonization of GF K/BxN mice with segmented filamentous bacteria (SFB) reinstated Th17 cell differentiation and the production of disease-inducing autoantibodies, and accelerated the onset of arthritis^28^. It was recently shown that SFB stimulate Th17 differentiation via induction of serum amyloid A (SAA) 1 and SAA2 production by intestinal epithelial cells^29,30^. IL-22 secretion by type 3 innate lymphoid cells (ILC3) was shown to be essential for SAA production by intestinal epithelial cells^30^. In addition, it was shown that inoculating GF arthritis-prone SKG mice with *Prevotella*-dominated microbiota of RA patients resulted in increased numbers of intestinal Th17 cells and enhanced the development of arthritis compared with mice receiving fecal microbiota from healthy controls^8^.

Importantly, depletion of intestinal microbiota with broad-spectrum antibiotics before the induction of antigen-induced arthritis and experimental autoimmune encephalomyelitis reduced disease severity^31,32^. A recent study showed that colonizing mice with the human gut commensal *Prevotella histicola* suppressed Th17 responses and the development of inflammatory arthritis in mice^33^. These observations suggest that microbiota-induced modulation of Th17 cell differentiation affects the initial development of inflammatory arthritis in mice. However, the role of the intestinal microbiota in the progression of established arthritis and the involved processes are unknown.

Using high-throughput sequencing of bacterial 16S rRNA, we here show that marked changes in the intestinal microbiota occur in the preclinical phase of inflammatory arthritis and precede the onset of the disease in mice. In addition, we show that modulation of intestinal microbiota during ongoing CIA alters both intestinal and joint-adjacent T cell profiles associated with changes in SAA and IL-22 expression, and can serve as a potential means to control the progression of established inflammatory arthritis.

## Results

### Altered composition of intestinal microbiota marks the preclinical phase of CIA and precedes the development of arthritis

**Figure 1:**
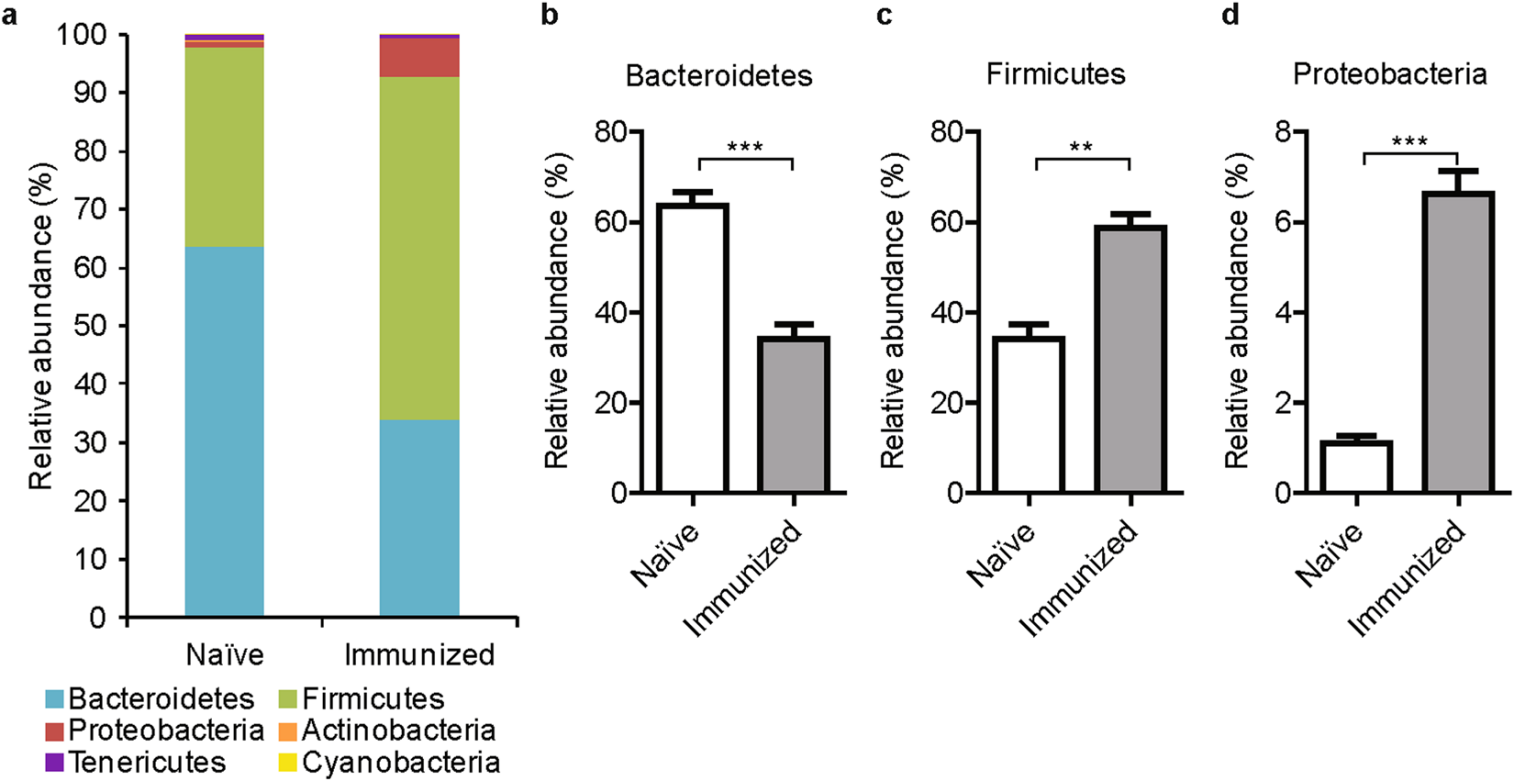
Altered composition of fecal microbiota after immunization with type II collagen. (**A**–**D**) 16S sequencing of fecal microbiota of naïve DBA mice just before immunization with type II collagen compared with microbiota of mice 21 days post-immunization before the booster injection and arthritis development. Relative abundances of bacterial taxa on phylum level is shown (N = 7 mice per group). Data are shown as mean + SEM. **p < 0.01, ***p < 0.001 by Mann-Whitney test followed by a correction for multiple testing using the Benjamini-Hochberg procedure.

To examine whether perturbations of intestinal microbiota precede or rather result from the inflammatory arthritis, we analyzed the intestinal microbiota of naïve DBA1/J mice before immunization with collagen type II (CII) and 21 days later prior to the booster injection using microbial 16S rRNA sequencing. Mice showed no macroscopic or histological signs of arthritis 21 days after immunization confirming the preclinical state (Supplementary Fig. S1A). We did not observe any significant changes in the number of operation taxonomic units (OTUs), the rarefaction curves of Chao1 index of bacterial richness, and the phylogenetic distance whole tree, a diversity metric based on both the number of observed OTUs and their phylogenetic distance (data not shown). Therefore, induction of CIA did not affect the intestinal bacterial richness and diversity. However, the preclinical, immune-priming phase of CIA was accompanied by marked compositional changes in intestinal microbiota at different taxonomic depths from phylum to genus. Analysis of the relative abundances at phylum level showed that the intestinal microbiota of naïve mice is dominated by the phylum Bacteroidetes (Fig. 1A,B). Comparison of the microbiota of naïve vs. immunized mice revealed that phylum Firmicutes dominates the intestinal microbiota in the preclinical phase of arthritis, during which the relative abundance of Bacteroidetes decreases (Fig. 1A–C). In addition, the relative abundance of Proteobacteria significantly increased after CIA immunization (Fig. 1D).

**Figure 2:**
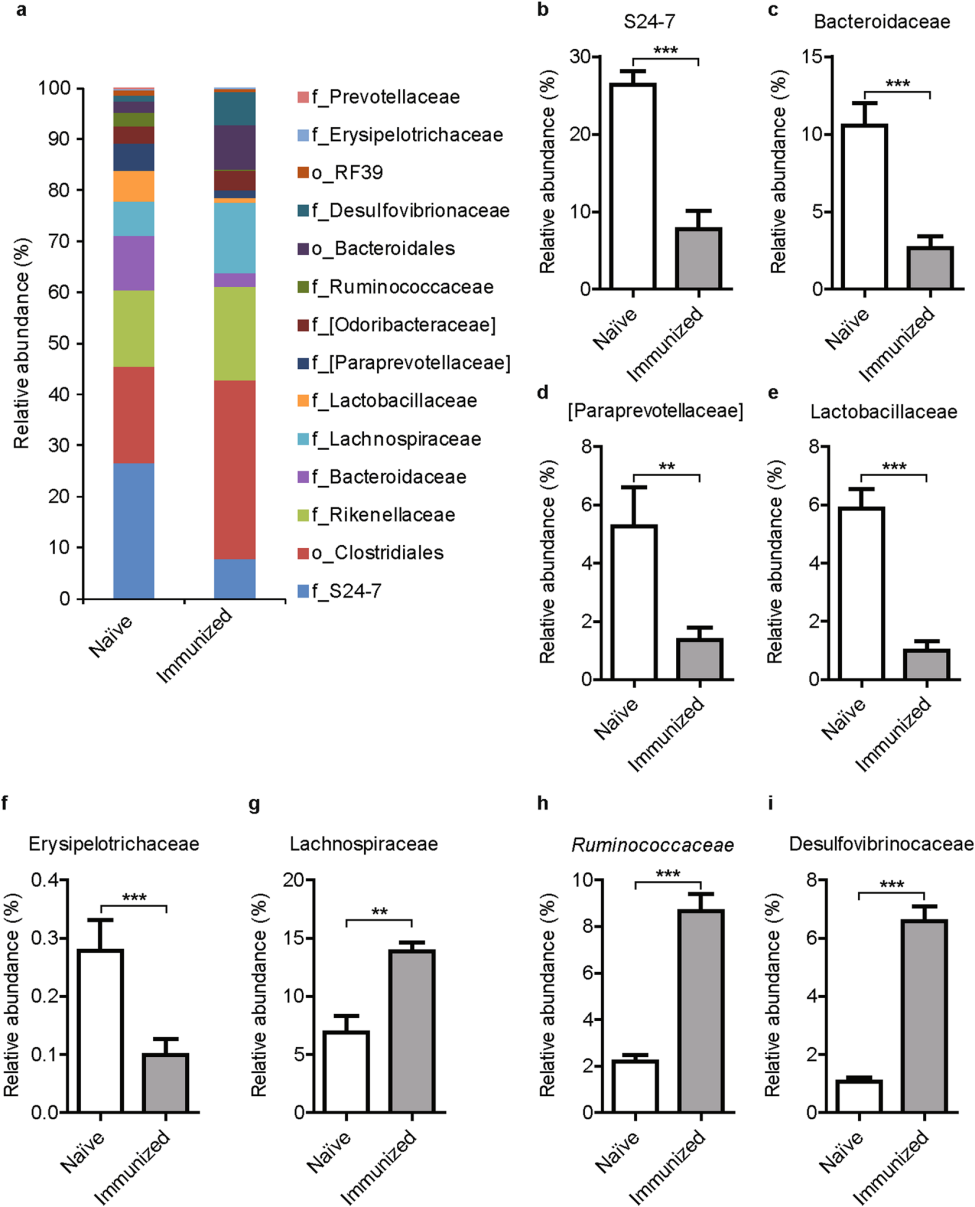
Intestinal microbiota undergoes marked changes in the preclinical phase of arthritis. (**A**) 16S sequencing of fecal microbiota of naïve DBA mice just before immunization with type II collagen compared with microbiota of mice day 21 post-immunization and before the booster injection. Relative abundances on family level is shown, however part of the bacteria could not be classified further than order-level, taxonomic level is indicated, o = order and f = family. (**B**–**I**) Relative abundances of intestinal bacteria at family level. N = 7 mice per group. Data are shown as mean + SEM. **p < 0.01, ***p < 0.001 by Mann-Whitney test followed by correction for multiple testing using the Benjamini-Hochberg procedure.

At the family level, we found that the families S24-7, Bacteroidaceae and Paraprevotellaceae in the phylum Bacteroidetes were significantly reduced in the immune-priming phase of CIA (Fig. 2A–D). Among Firmicutes, the families Lachnospiraceae and Ruminococcaceae were expanded while Lactobacillaceae and Erysipelotrichaceae were reduced during the preclinical phase of arthritis (Fig. 2A,E–H). Furthermore, the family Desulfovibrionaceae in the phylum Proteobacteria was significantly increased in immunized compared with naïve mice (Fig. 2I). After correction for multiple testing using the Benjamini-Hochberg procedure, we observed a significant increase in the relative abundance of the genera *Oscillospira* and *Ruminococcus* (both in Ruminococcaceae family) and a significant decrease in *Bacteroides* (family Bacteroidaceae), *Prevotella* (family Paraprevotellaceae), and *Lactobacillus* (family Lactobacillaceae) (Supplementary Fig. S2). Only a few OTUs could be assigned at the species level, including two OTUs assigned to *Lactobacillus reuteri* that were significantly reduced, and two OTUs aligned to the species *Ruminococcus gnavus* that were significantly increased in preclinical phase of CIA (Supplementary Table S1). Altogether, our data strongly suggest that alterations of the intestinal microbiota occur in the preclinical, induction phase of inflammatory arthritis and likely precede the clinical manifestations of the disease.

### Immunization with type II collagen results in increased serum SAA levels and local Th17 cell frequency in the preclinical phase of arthritis

As mentioned earlier, no signs of arthritis could be observed on macroscopic or histological level (Supplementary Fig. S1A). Serum amyloid A (SAA) has been shown to be upregulated at early stages of inflammatory arthritis. Therefore, we measured SAA protein levels in serum along with the gene expression of SAA isoforms in the synovium of naïve mice in comparison with immunized mice at day 21 post-immunization before the onset of arthritis. SAA levels in serum showed a significant increase 21 days after immunization before the onset of arthritis compared to naïve condition (Supplementary Fig. S1B). In addition, we observed a non-significant increase of SAA3 expression in the knee synovial tissue (p = 0.2345). However, SAA1 and SAA2 were expressed at low levels and remained similar to the naïve condition (Supplementary Fig. S1C). In addition, we analyzed alterations of the T helper cell response in joint-draining lymph nodes in the pre-clinical phase of CIA. Flow cytometry analysis showed that the percentage of Th17 cells was significantly higher in immunized (preclinical) mice compared to naïve mice (Supplementary Fig. S1D). The percentages Th1 and Treg cells and the absolute numbers of Th1, Th17 and Treg cells were not affected by immunization (Supplementary Fig. S1A and E). These data suggest that immune activation characterized by increase in SAA serum levels and local Th17 activation precedes the development of CIA.

### Treatment with broad-spectrum antibiotics results in partial elimination and compositional shifts in the intestinal microbiota of CIA mice

GF condition and elimination of microbiota before the induction of arthritis affects the intestinal T cell balance and initiation of arthritis^26–28,32^. To examine whether the gut microbiota modulate T cell responses and disease progression during established arthritis, we treated mice with ongoing CIA with broad-spectrum antibiotics (ABX) previously shown to alter the abundance and composition of intestinal bacteria in naïve mice^31,34^. To determine the effects of antibiotic-treatment during CIA on the intestinal microbiota, we analyzed bacterial 16S rRNA sequences of ABX-treated and control CIA mice. Antibiotic treatment strongly reduced the number of 16S sequence reads compared with the control treatment (mean of 9002 ± 2167 versus 29392 ± 1140 reads, respectively; p = 0.004). In addition, antibiotic treatment resulted in reduced diversity and a marked compositional shift in the remaining intestinal bacterial communities of the CIA mice (Supplementary Fig. S3). Specifically, almost all bacteria belonging to the phylum Bacteroidetes were eliminated (similar to response in humans^35^), and the majority of the bacteria that remained after antibiotic treatment belonged to the phylum Firmicutes (Supplementary Fig. S3A). The relative abundances of intestinal bacterial taxa of the control and antibiotic-treated CIA mice at family and genus levels are shown in Supplementary Fig. S3B and C.

Since SFB have been reported to trigger the development of autoimmunity in mice^28,29,36^, we specifically examined the presence of SFB. We detected SFB (family Clostridiaceae; genus *Candidatus Arthromitus*) in the 16S sequences of only 2 out of 10 CIA mice before the ABX-treatment. After treatment SFB 16S sequences were detectable in 2 out of 5 untreated control mice but none of the ABX-treated mice. Using SFB-specific primers for qPCR^37^, we could confirm the presence of SFB in all cages of CIA mice before the start of antibiotic treatment. At the end point, while we could still detect SFB in the untreated control group of CIA mice (Ct ≥ 32), SFB were not detectable in any of the ABX-treated CIA mice (data not shown).

### Partial elimination of intestinal microbiota during ongoing CIA suppresses proinflammatory T helper cell subsets in intestinal lamina propria

**Figure 3:**
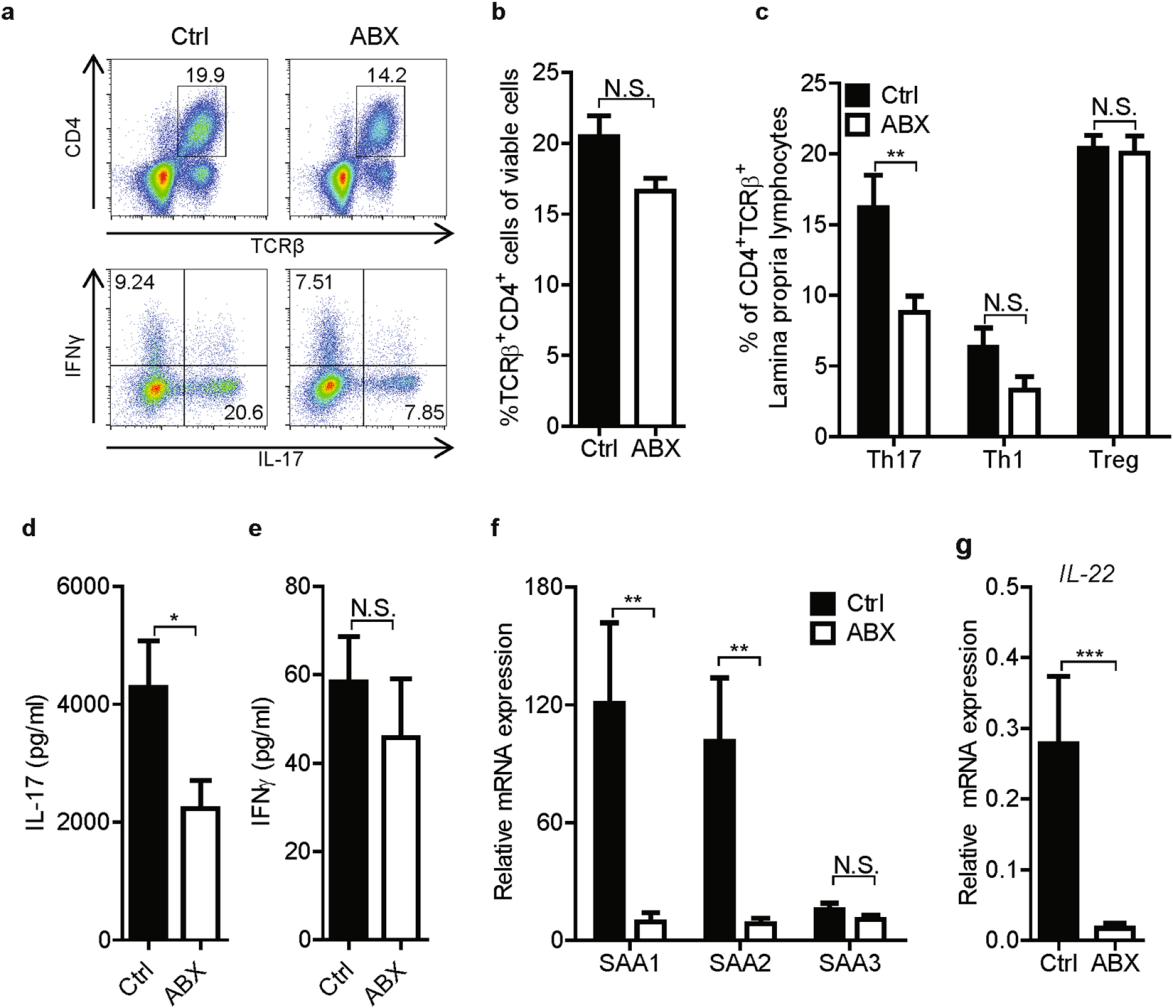
Treatment with broad-spectrum antibiotics during established CIA skews the intestinal T helper cell balance. (**A**) Representative flow cytometry plots showing the percentage of Th1 and Th17 cells per gate. (**B**) Percentage of CD4^+^TCRβ^+^ Th cells of viable cells isolated from the intestinal lamina propria. (**C**) Percentage of IL-17^+^ (Th17), IFNγ^+^ (Th1) and FoxP3^+^ (Treg) cells among CD4^+^TCRβ^+^ cells isolated from intestinal lamina propria. (**D**,**E**) Production of IL-17 and IFNγ by lamina propria lymphocytes upon *ex vivo* stimulation with PMA and ionomycin for 5 hours, measured by Luminex cytokine array. (**A**–**E**) Cells were isolated from untreated control (Ctrl, n = 12) and antibiotic-treated (ABX, n = 10) mice at the end of the antibiotic treatment. (**F**,**G**) Gene expression of serum amyloid A (SAA) 1, 2 and 3 (**F**) and IL-22 (**G**) in terminal ileum of small intestine. Gene expression was measured by qPCR in tissues from Ctrl (n = 18) and ABX (n = 19) mice. Relative mRNA expression is shown as 2^−ΔCt^ *10000, corrected for GAPDH. Data are shown as mean + SEM. *p < 0.05, **p < 0.01, ***p < 0.001 by Mann-Whitney test; N.S. not significant.

It has been described that partial depletion of intestinal microbiota in naïve mice results in reduced production of interferon γ (IFNγ) and IL-17 by mucosal CD4^+^ T cells in small intestine lamina propria (LP)^34^. Therefore, we investigated whether depletion of intestinal microbiota during CIA affects the intestinal T helper cell balance. Flow cytometry analysis revealed a slight, non-significant reduction in the proportion of TCRβ^+^CD4^+^ T helper cells in intestinal LP after ABX treatment (p = 0.06, Fig. 3A,B). Antibiotic treatment resulted in a specific reduction in the percentage of TCRβ^+^CD4^+^IL-17^+^ Th17 cells in LP of mice with CIA (p = 0.004, Fig. 3A and C), similar to previously published observations in naïve mice^34^. In addition, a reduction in TCRβ^+^CD4^+^IFNγ^+^ Th1 cells was observed after ABX treatment; however, this effect was heterogeneous and did not reach statistical significance (p = 0.1059, Fig. 3A and C). In contrast, ABX treatment did not affect the proportion of TCRβ^+^CD4^+^Foxp3^+^ Treg cells in the intestinal LP of CIA mice (Fig. 3C).

Next, we analyzed cytokine production by LP lymphocytes (LPLs) stimulated *ex vivo* with PMA and ionomycin. In accordance with the flow cytometry data, IL-17 production was significantly reduced in LPLs of ABX-treated mice compared with control mice (Fig. 3D). IL-4 showed a trend towards reduction (p = 0.0892; Supplementary Fig. S4A), while the production of IFNγ and IL-10 remained unaffected (p = 0.2428 and p = 0.9705, respectively; Fig. 3E and Supplementary Fig. S4B). Analysis of the gene expression showed that ABX treatment significantly reduced expression of IL-17A mRNA in terminal ileum (p = 0.008), a main site for microbiota-induced T cell modulation, whereas the expression IFNγ and IL-10 mRNA was not affected significantly (Supplementary Fig. S4C). The expression of IL-17, IFNγ and IL-10 in colon tissue was not affected by antibiotic treatment (Supplementary Fig. S4D) In addition, while unaffected in small intestine, the expression of the Treg-related transcription factor FoxP3 was significantly upregulated in the colon of ABX-treated mice (Supplementary Fig. S4E). Altogether, these observations indicate that partial elimination of intestinal microbiota during established CIA modulates the mucosal T helper cell balance mainly presented by a suppression of Th17 cells.

### Antibiotic treatment reduces intestinal SAA1 and SAA2 expression in CIA mice

The mechanism of microbiota-induced Th17 cell differentiation in intestinal LP was reported for SFB as model organisms. It was demonstrated that SFB-induced intestinal epithelial production of SAA1 and SAA2 in terminal ileum, stimulated by ILC3-derived IL-22, is required for the functional differentiation of Th17 cells and the production of IL-17^30^. Therefore, we analyzed the expression of SAA1, SAA2 and SAA3 isoforms as well as IL-22 in terminal ileum. In line with the reduction of Th17 cell abundance in LP upon ABX treatment (Fig. 3A and C), both SAA1 and SAA2 were markedly diminished in terminal ileum of ABX-treated CIA mice compared with the control CIA mice (Fig. 3F). However, SAA3 expression was lower than SAA1 and SAA2, and showed a non-significant reduction (Fig. 3F). Furthermore, while IL-22 was detectable in the majority of control CIA mice (15 out of 18), its expression was markedly attenuated in ABX-treated CIA mice and was detectable only in 2 out of 19 mice (Fig. 3G). This suggests that reduced Th17 cell differentiation and IL-17 production in ABX-treated CIA mice may have resulted from lower IL-22-mediated induction of SAA1 and SAA2 due to the elimination of commensal microbiota.

### Elimination of intestinal microbiota reduces the severity of established T cell-mediated experimental arthritis

**Figure 4:**
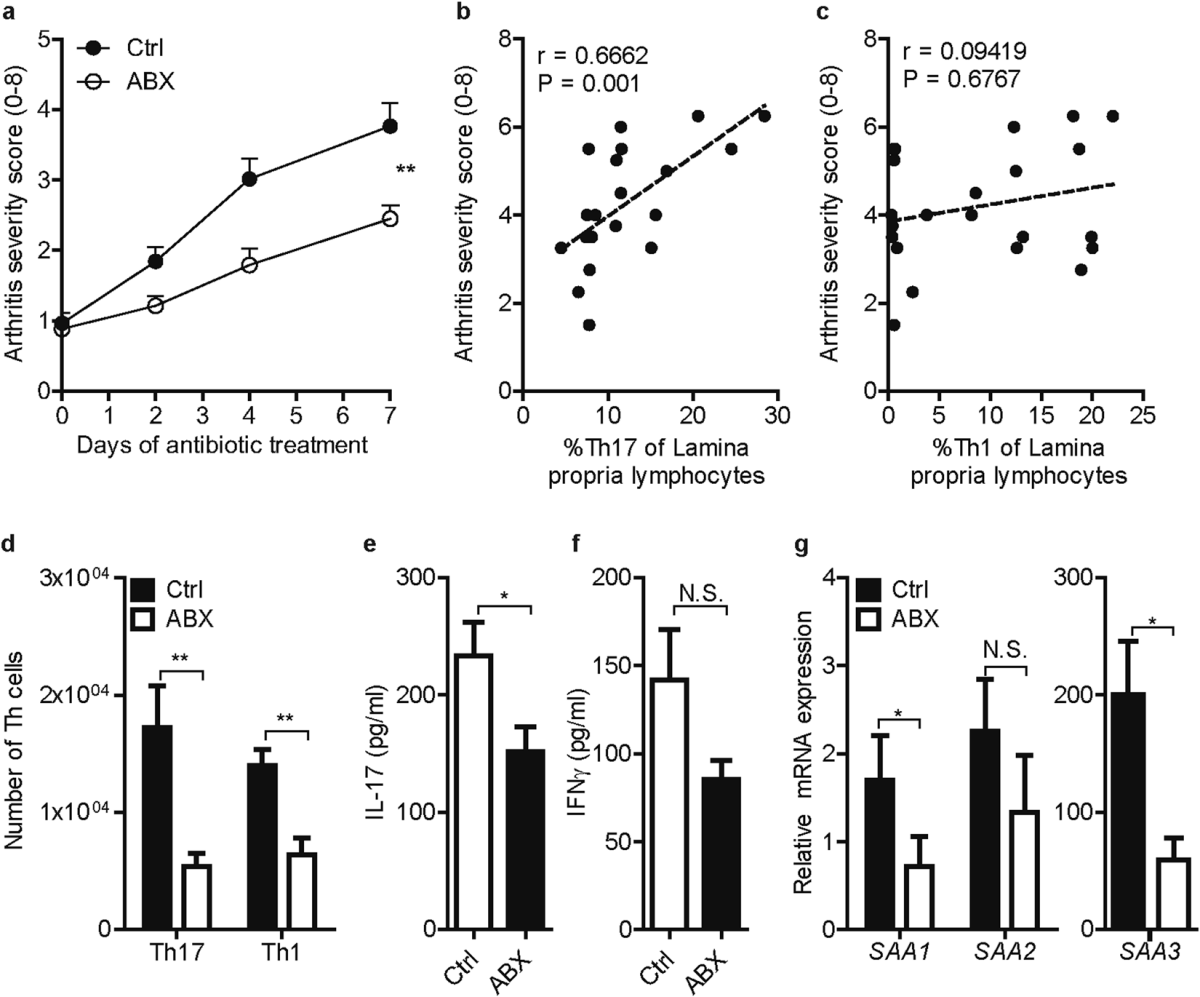
Elimination of intestinal microbiota reduces the severity of established T cell-mediated experimental arthritis. (**A**) Arthritis severity scores of untreated control (Ctrl) and antibiotic-treated (ABX) CIA mice; n = 19 mice per group from two independent experiments. Severity was scored on a scale of 0–2 for each paw. **p < 0.01 by two-tailed Mann-Whitney U test. (**B,C**) Correlation between the percentages of Th17 and Th1 cells in the intestinal lamina propria and arthritis severity scores of mice with CIA. The Spearman’s correlation coefficient (r) and the p value are shown at the top left of the graphs. (**D**) Number of Th17 (CD4^+^TCRβ^+^ IL-17^+^) and Th1 (CD4^+^TCRβ^+^IFNγ^+^) cells isolated from popliteal lymph nodes (pLN) of Ctrl (n = 11) and ABX-treated (n = 10) CIA mice. (**E,F**) Concentration of IL-17 and IFNγ in culture supernatants of pLN cells from Ctrl (n = 12) and (n = 11) ABX mice *ex vivo* stimulated with PMA and ionomycin for 5 hours. (**G**) Gene expression of SAA1, SAA2 and SAA3 in synovial biopsies of Ctrl (n = 12) and ABX (n = 13) mice. Relative mRNA expression is shown as 2^−ΔCt^ *10000, corrected for GAPDH. (**D–G**) Data are shown as mean + SEM. *p < 0.05, **p < 0.01 by Mann-Whitney test.

We monitored the severity of arthritis in control and antibiotic-treated CIA mice. ABX treatment significantly attenuated the severity of established arthritis in CIA mice (Fig. 4A). Interestingly, the abundance of Th17 cells in the intestinal LP of CIA mice showed a striking correlation with the severity of arthritis (r = 0.666; p = 0.001), while no correlation was found between the LP Th1 cells and arthritis (Fig. 4B and C). This supports the presence of a potentially relevant link between the microbiota-induced intestinal Th17 cells and the severity of experimental arthritis. Importantly, the numbers of Th17 and Th1 cells in joint-draining pLN were significantly reduced in ABX-treated CIA mice compared with control mice (Fig. 4D). Accordingly, pLN cells from ABX-treated mice produced less IL-17 (p = 0.0392) upon *ex vivo* stimulation with PMA and ionomycin (Fig. 4E). The release of IFNγ in *ex vivo* LPL cultures was not significantly affected by the ABX treatment (p = 0.3390, Fig. 4F). Furthermore, the production of IL-10 and IL-4 remained unaffected (p = 0.5179 and p = 0.4417 respectively, data not shown). These observations suggest that partial elimination of intestinal microbiota after the onset of arthritis, i.e. during established CIA, attenuates the disease and is associated with a parallel reduction in IL-17 production in joint-draining lymph nodes and intestinal LP. We further assessed the expression of the SAA isoforms in synovial tissue. In contrast to terminal ileum, SAA3 was the major isoform in the synovium and its expression was strongly decreased in antibiotic-treated CIA mice (p = 0.0180, Fig. 4G). Furthermore, reduced joint inflammation in antibiotic-treated CIA mice was accompanied by a significant suppression of SAA1 (p = 0.0471) and a non-significant reduction of SAA2 (p = 0.0771) mRNA expression in synovial tissue (Fig. 4G).

Interestingly, while both LP and pLN Th17 cells were affected, we could not find any difference in serum levels of anti-mouse collagen type II auto-antibodies between the control and ABX-treated mice (Supplementary Fig. S5). Therefore, the observed reduction in CIA disease severity by post-onset antibiotic treatment is not associated with a suppression of CII-specific antibody response.

In addition, elimination of intestinal microbiota by the ABX treatment was unable to modulate the severity of serum-transfer arthritis, a T cell-independent model^38,39^ induced by the transfer of arthritogenic K/BxN serum (Supplementary Fig. S6). These data suggest that elimination of intestinal microbiota during T cell-mediated experimental arthritis can attenuate Th17-driven disease processes, whereas arthritis induced by the already generated arthritogenic antibodies does not depend on the intestinal microbiota.

## Discussion

Several studies have shown that the composition of intestinal microbiota is perturbed in patients with inflammatory arthritis such as new-onset as well as chronic RA^5–7,9,40^. The development of autoimmunity in RA starts several years before the appearance of the clinical signs, a process that commensal microbiota may be able to promote^41–43^. Therefore, it is important to understand whether the observed perturbations in the intestinal microbiota precede the onset of inflammatory arthritis or are merely a consequence of disease. While this remains to be determined in humans at-risk of developing inflammatory arthritis, we here show that in experimental arthritis, marked changes in the intestinal microbiota occur in the preclinical immune-priming phase and precede the onset of inflammatory arthritis. A recent study showed that the composition of microbiota prior to arthritis onset differs between the CIA–susceptible and –resistant mice^44^. This study found that the family Lactobacillaceae was more abundant in CIA-susceptible mice, whereas Desulfovibrionaceae and Lachnospiraceae were more abundant in CIA–resistant mice^44^. Moreover, by comparing the fecal bacterial composition in the immunized mice before and after the onset of arthritis, it was shown that the families Bacteroidaceae, Lachnospiraceae and S24-7 significantly increased as arthritis initiated^44^. Our studies focused on the preclinical phase of arthritis and show that this phase is characterized by decreased S24-7 and Bacteroidaceae and increased Ruminococcaceae, Lachnospiraceae and Desulfovibrinocaceae. Increased Lachnospiraceae has previously also been demonstrated in RA patients with varying disease duration^7^. Our data demonstrate that the increase in Lachnospiraceae occurs before the onset of arthritis and is induced by the immunization during the preclinical phase. Another study compared the microbiome of healthy non-immunized mice and mice with CIA. This study found that Clostridiales, Lachnospiraceae and *Ruminoccocus gnavus* were more abundant in the mice with established CIA compared to the healthy mice^45^. Interestingly, we found these taxa to be increased in the preclinical phase and before the onset of arthritis. Altogether, these observations suggest that early perturbations of the intestinal microbiota during the immune-priming phase may contribute to the initiation of inflammatory arthritis.

We previously showed that treatment with Tobramycin resulted in a near-complete elimination of the genera *Helicobacter* and *Flexispira* (both belonging to the family Helicobacteraceae) and suppressed arthritis in IL-1Ra-deficient mice^46^. In addition, a strong and highly significant reduction in the genus *Clostridium* was observed, which suggests that these bacteria may promote intestinal Th17 cell differentiation and arthritis^46^. In the present study, we observed a strong and significant increase in the order Clostridiales after immunization with collagen. However, a large part of the order Clostridiales could not be assigned to a higher taxonomic level and the exact effects on the genus *Clostridium* remain to be clarified.

Previous studies showed that *Prevotella copri* is expanded in patients with new-onset RA^6,8^. In addition, it was shown that transfer of *Prevotella*-dominated microbiota of RA patients to SKG mice results in increased numbers of intestinal Th17 cells and exacerbates arthritis compared with mice receiving fecal microbiota from healthy controls^8^. Identification of the exact *Prevotella* species altered upon immunization in CIA mice was not possible using 16S rRNA gene sequencing due to inherently limited resolution of this technique. Furthermore, none of the observed OTUs related to *Prevotella* in our database could be assigned to *P. copri*. Therefore, we performed *P. copri*-specific qPCR using species specific primers^6^ to detect *P. copri* with a higher sensitivity (primer sequences in Suppl. Table S2). While our positive control (DNA isolated from anaerobically grown *P. copri*) showed a positive signal on qPCR with a Ct value of 17.7 cycles, *P. copri* DNA was detected at very low levels (Ct value of >33 cycles) in fecal DNA of the mice and was not different between naïve and immunized mice. Therefore, the abundance of *P. Copri* was not altered in our mice by CII immunization.

Loss of intestinal microbial diversity and richness coincides with autoimmune diseases such as diabetes, RA and PsA in patients^5,6,47^. Our data suggest that the preclinical phase of experimental arthritis is not accompanied by significant changes in the microbial diversity and richness. It is tempting to speculate that the reduced microbial diversity reported in human autoimmune disease may be a consequence of the ongoing inflammation at the time of sampling. While our study was able to identify robust changes in the intestinal microbiota that precede the onset of inflammatory arthritis, it is important to expand and strengthen such studies with shotgun sequencing coupled with transcriptomics and metabolomics approaches to understand the full functional capacity of the microbiome during the preclinical phase of arthritis. More importantly, similar studies are warranted in humans at risk of developing autoimmune arthritis before the onset of inflammation to unravel the relevance of the microbiome in predisposition or the inductive phase of the disease.

Intestinal LP is a major source of Th17 cells in the body^29,48^. Previous studies have shown that alterations of the gut commensal bacteria have strong effects on Th17 cell differentiation^29,48^. Commensal microbiota have been shown to promote the initiation of spontaneous arthritis in IL-1Ra^−/−^ mice as well as in K/BxN mice, which bear a transgenic auto-reactive T cell receptor^27,28^. In non-transgenic animal models, treatment with a cocktail of vancomycin, neomycin and metronidazole before the induction of antigen-induced arthritis resulted in milder disease^32^. On the other hand, gut microbiota-induced IL-1 and IL-6 can trigger the respective cytokine receptors on B cells to promote differentiation of IL-10-producing regulatory B cells and restrict antigen-induced arthritis^32^. However, it is not clear whether modulation of intestinal microbiota after the onset of arthritis can still alter the T cell phenotype and modify the progression of arthritis.

In this study, we employed a widely-used model of inflammatory arthritis induced in non-transgenic mice to assess the role of intestinal microbiota during already established disease. We show here for the first time that elimination of intestinal microbiota during established CIA attenuates inflammatory arthritis. This is in line with earlier reports that oral antibiotic treatments with sulfasalazine and minocycline reduce RA disease activity in patients with disease duration of <1 year^49,50^. Elimination of microbiota after the onset of arthritis resulted in suppression of intestinal SAA1, SAA2 and IL-22 expression and a specific reduction of LP Th17 cells accompanied with reduced Th17 cell abundance in joint-draining lymph nodes of the CIA mice. The potential of intestinal T cells to migrate to peripheral lymph nodes, as reported before, may explain these observations^51^. Our data also show for the first time a direct correlation between the abundance of LP Th17 cells and the severity of arthritis, which further supports a continued pro-inflammatory gut-joint axis during established arthritis.

Previous studies identified SFB as potent inducers of Th17 cell differentiation promoting arthritis development in the T cell-mediated K/BxN model^28,29^. Elimination of SFB by ABX treatment in our study is consistent with reduced LP Th17 cells and suppressed expression of SAA1 and SAA2, which were recently reported to act on Th17 cells to further enhance their differentiation and effector function^30^. Our data suggest that the observed Th17 cell reduction upon antibiotic treatment in our study is at least partially due to the elimination of SFB, although the effect of other microbiota cannot be excluded. Interestingly, although SAA1 and SAA2 were the most abundant SAA isoforms in the intestine, SAA3 appeared to be the prominent isoform in the target tissue, i.e. synovium, and its expression was similarly affected by the microbiota. While SAA1/SAA2 were shown to promote intestinal Th17 cells, it is not known if SAA has a similar effect on Th17 cells residing in the synovium. Several studies have shown the relevance of SAA as a marker of RA disease activity and its association with disease-relevant autoantibodies and acute phase proteins^52–55^. In addition, several RA therapies have been shown to influence circulating SAA levels during treatment^52–55^. Furthermore, SAA is also a good indicator of cardiovascular and renal involvement in patients with RA^56^. Besides being a valuable marker for disease activity, SAA has been shown to mediate inflammatory and angiogenic mechanisms, likely through TLR2^57,58^. Our data demonstrate that increased systemic level of SAA precedes the onset of arthritis and can be used as a disease marker even before its clinical onset.

In line with the essential role of IL-22 in intestinal SAA production^30^, we observed a significant reduction of intestinal IL-22 expression after antibiotic treatment. IL-22 was shown to be highly expressed in synovium of RA patients and serum levels of IL-22 are significantly higher in RA patients compared with healthy controls and correlate with disease activity^59,60^. In addition, elevated levels of IL-22 in early disease seem to predict erosive progression, suggesting that IL-22 has a role in the pathophysiology of RA^61^. In patients with PsA, IL-22-producing CD4^+^ T cells were increased in peripheral blood as well; however, IL-22-positive cells could not be detected in synovial fluid and tissue of PsA patients^62^.

Although our studies support suppression of intestinal and joint-adjacent Th17 differentiation as an underlying mechanism for attenuation of arthritis upon elimination of intestinal microbiota, involvement of microbiota-induced regulation of other immune cells is not excluded. In this regard, a recent study showed that continuous treatment with ampicillin and vancomycin limited follicular T helper (Tfh) cell differentiation and germinal center formation in K/BxN mice, resulting in reduced production of arthritogenic autoantibodies and less severe arthritis^63^. These effects appeared to be independent of IL-17, which was found to be dispensable for the disease development in this study, although an earlier study showed that IL-17 blockade could reduce anti-GPI antibody levels and suppress K/BxN arthritis^28,63^. We observed no effect of ABX treatment on serum levels of anti-mouse collagen type II antibodies in our experiments, suggesting that during ongoing CIA, intestinal microbiota affect Th17 cell differentiation and arthritis without influencing the autoantibody response. Therefore, intestinal microbiota appear to influence different disease mechanisms depending on the disease phase and processes being studied. In addition, by mimicking the effector phase of the K/BxN arthritis using KRN serum-transfer model, we show that intestinal microbiota do not play an important role in K/BxN arthritis anymore once the disease-inducing auto-antibodies have been developed (Supplementary Fig. S6).

In agreement with our findings, IL-17 producing T cells can promote arthritis independent of their influence on antibody production, since transfer of IL-17-producing KRN transgenic T cells into a B-cell-deficient host can enhance arthritis in an IL-17-dependent manner^64^. In fact, Th17 cells contribute to several pro-inflammatory and tissue-destructive processes during inflammatory arthritis and may represent a relevant target to control the disease^12,13^. The role of Th17 cells in arthritis is likely to expand beyond its prototypic cytokine IL-17A. The actual requirement of Th17 cells for the microbiota-induced aggravation of arthritis requires, however, further investigation.

It has been described that Th17 cells can transition into a stage in which they produce both IL-17 and IFNγ, after which they may lose their expression of IL-17^65^. A recent study showed that these IFNγ-producing cells, called ex-Th17 cells or non-classical Th1 cells, are not constrained by regulatory T cells^66^. In addition, these cells were shown to accumulate in arthritic joint of arthritis patients^66^, suggesting a role in the pathogenesis of RA. We analyzed the proportions of IL-17^+^ IFNγ^+^ double-positive TCRβ^+^CD4^+^ cells. We observed a non-significant reduction in the percentage, but not absolute number, of IL-17^+^ IFNγ^+^ TCRβ^+^CD4^+^ in intestinal lamina propria by antibiotic treatment (Mean ± SEM of control: 1.049% ± 0.2594; ABX: 0.4699% ± 0.1297). Furthermore, while there was no significant effect of antibiotic treatment on the percentage of IL-17^+^ IFNγ^+^ TCRβ^+^CD4^+^ cells in pLNs, we observed a non-significant reduction in the numbers of these cells (Ctrl: 537.9 ± 228.6; ABX: 240.6 ± 76.38). We cannot exclude the possibility that the Th1 cells, which were significantly reduced in pLNs after ABX-treatment, also included exTh17 cells only producing IFNγ.

Multiple studies showed that Th17 levels are high in treatment-naïve patients with early RA^67–69^. While elevated levels of Th17 cells have been found in RA and PsA patients, reports on the proportion of Th17 cells in patients with established RA are inconsistent^62,67,70–75^. Based on the higher efficacy of IL-17 blockade in PsA compared with RA, it is plausible that the PsA microbiome has a higher potential to induce mucosal Th17 and IL-17 responses. On the other hand, it is possible that stratification of patients with RA based on their gut microbiota and its Th17-inducing potential improves the efficacy of IL-17 inhibition in RA. A side-by-side comparison of matched cohorts of PsA and RA patients will be required to address the exact differences between RA and PsA microbiomes and their relevance for the efficacy of biologic treatments.

In summary, our study suggests that perturbations in the intestinal microbiota precede the onset of arthritis. In addition, we show that elimination of intestinal microbiota during established CIA reduces Th17 cell abundance in intestinal mucosa and joint-draining lymph nodes and attenuates arthritis without affecting autoantibody production. While our study does not advocate the use of antibiotics as a treatment for RA or related inflammatory arthropathies, it supports the notion that inflammatory signals provided by the gut microbiota continue to promote arthritis after its onset. The striking correlation between the abundance of intestinal Th17 cells and the severity of arthritis supports this link. Understanding the exact mechanisms linking the intestinal T cell response with arthritis may help identifying novel therapeutic strategies for inflammatory arthritis.

## Material and Methods

### Mice and induction of CIA

Male DBA/1J mice (Janvier, France) were housed in individually-ventilated cages, and water and food were provided *ad libitum*. CIA was induced by intradermal injection of 100 µg bovine type II collagen (CII) in Freund’s complete adjuvant on day 0 and intraperitoneal booster injection of 100 µg CII in PBS on day 21. Clinical onset and progression of arthritis was scored on a scale between 0–2 for each paw as described before^76^. All animal studies were approved by the institutional review board (Animal Experimentation Committee of Radboud University Medical Center) and were conducted in accordance with the institutional guidelines.

### Sample collection and DNA extraction

Feces were collected and stored at −80 °C until processing. Fecal DNA was collected using the PowerLyzer DNA isolation kit (MO BIO laboratories) following manufacturer’s instructions.

### 16S sequencing of intestinal microbiota

Amplicon library preparation was performed using an automated platform (Biomek 4000) with a custom liquid handling method. For each sample, the V4 region of the bacterial 16S rRNA gene was amplified in duplicate reactions using primer set 515F/806R, which nearly universally amplifies bacterial and archaeal 16S rRNA genes. Each unique barcoded amplicon was generated in pairs of 25 μl reactions with the following reaction conditions: 11 μl PCR-grade H2O, 10 μl Hot MasterMix, 2  μl of forward and reverse barcoded primer (5 μM) and 2 μl template DNA. Reactions were run on a C1000 Touch Thermal Cycler (Bio-Rad) with the following cycling conditions: initial denaturing at 94 °C for 3 min followed by 35 cycles of denaturation at 94 °C for 45 seconds, annealing at 58 °C for 1 minute, and extension at 72 °C for 90 seconds, with a final extension of 10 minutes at 72 °C. Amplicons were quantified using the Agilent 2200 TapeStation system and pooled. Purification was then performed using Ampure XT (Beckman Coulter) as per the manufacturer instructions. Sequencing was performed using the MiSeq (Illumina) platform to produce 150 base-pair end reads and define the microbiota composition, as we previously described^77^. The obtained 16S rRNA sequences were analyzed using the Quantitative Insights into Microbial Ecology (QIIME) pipeline for analysis of community sequence data. Briefly, reads were demultiplexed and quality filtered with default parameters using prinseq. Sequences were then clustered into operational taxonomic units (OTUs) using a 97% similarity threshold with USEARCH and the Greengenes 16S reference dataset and taxonomy^78^. PyNAST was used as the default alignment tool for QIIME.

### Antibiotic treatment

CIA mice with a macroscopic score of 0.25–2.75 were randomized for treatment with a cocktail of 0.5 g/L vancomycin (Sigma), and 1 g/L metronidazole (Acros Organics), neomycin trisulfate (Sigma) and ampicillin sodium salt (Sigma) provided in drinking water containing 6 g/L sucrose for 1 week or for the control treatment (only sucrose).

### Isolation of lamina propria-lymphocytes (LPLs)

Small intestine and colon were isolated and residual mesenteric fat and Peyer’s patches were removed. The intestine was opened longitudinally, washed with ice-cold PBS and treated twice with 5 mM EDTA for 10 minutes at 37 °C to remove the epithelial cells. The tissue was further digested using collagenase D (0.5 mg/ml, Roche), DNase I (0.25 mg/ml, Sigma), and Dispase (50 U/ml, Fisher) for 3 cycles of 20 minutes at 37 °C. Cells were passed through a 40 μM cell strainer and LP lymphocytes were harvested at the interphase of a 40%/80% Percoll gradient (Sigma).

### Induction of K/BxN serum-transfer arthritis

Serum-transfer arthritis was induced in C57Bl/6 mice by two intraperitoneal injections of 200 µl arthritogenic serum derived from arthritic K/BxN mice on days 0 and 2, as described before^79^. Mice were treated with antibiotics as described above for CIA starting from day 0 because of the acute nature and short span of the serum-transfer model.

### Flow cytometry

Cells were incubated with phorbol 12-myristate 13-acetate (PMA; 50 ng/ml; Sigma), ionomycin (1 μg/ml; Sigma), and Brefeldin A (1 μl/ml; BD Biosciences) at 37 °C for 4 hours and stained with anti-CD4-APC (Biolegend), anti-TCRβ-FITC (Biolegend) and fixable viability dye (eBioscience). For intracellular staining, cells were fixed and permeabilized using fixation/permeabilization buffer (eBioscience) and stained with anti-IL-17-PECy7 (biolegend), anti-IFNγ-PE (BD pharmingen), and anti-FOXP3-PE (eBioscience) in permeabilization buffer (eBioscience). Data were collected on Gallios flow cytometer (Beckman Coulter) and analyzed with Flowjo 10.0 software.

### Cell culture and measurement of cytokines

Popliteal lymph nodes (pLN) were disrupted on a 70 µm cell strainer. LPLs (4 × 10^5^ cells/well) and pLN cells (2 × 10^5^ cells/well) were cultured with PMA (50 ng/ml; sigma) and ionomycin (1 µg/ml; Sigma) for 6 hours. Cytokine concentrations in culture supernatants and mice sera were measured by Luminex using the mouse cytokine/chemokine magnetic bead kits (Bio-Rad).

### RNA isolation and quantitative real-time PCR

Tissues were homogenized using a MagNA lyser instrument (Roche). RNA was isolated in TRIzol reagent (Sigma) as described before^27^. Quantitative real-time PCR (qRT-PCR) was performed using the StepOne System (Applied Biosystems) using the SYBR green Master Mix (Applied Biosystems). Primer sequences are shown in Supplementary Table S2.

### Measurement of anti-collagen antibodies

Anti-mouse CII IgG1 and IgG2a antibodies were determined in serum using ELISA, as reported before^80^. Briefly, 96 wells plates were coated with 0.1 µg of mouse type II collagen (chondrex). Non-specific binding sites were blocked by a 5% solution of milk powder. Serial dilutions of mouse sera were added, followed by incubation with peroxidase-labeled isotype-specific goat-anti-mouse-IgG antibodies and 5-aminosalicylic acid as substrate. Absorbance was measured at 450 nm.

### SAA serum levels

Serum amyloid A in serum was measured with ELISA (R&D systems) according to the manufacturer’s guidelines.

### Histology

Total ankle joints were isolated and fixed in 4% formaldehyde for 4 days, thereafter decalcified in 5% formic acid and embedded in paraffin. Tissue sections of 7 μM were stained using hematoxylin and eosin to study synovial inflammation, cartilage destruction, and bone erosion.

### Statistics

Differences in the relative abundance of bacterial taxa between groups were evaluated using Mann-Whitney test. Differences in operational taxonomic units (OTUs) were evaluated using two-tailed Student’s t-test. We corrected for multiple testing using the Benjamini and Hochberg procedure with false discovery rate (FDR) set at 10%, and differences with a p-value < 0.05 which passed the FDR test were considered statistically significant. Mann-Whitney U test was used to compare cell and cytokine levels between treatment groups. For arthritis scores, two-tailed Mann-Whitney U test was performed for area under the curve. Correlations were examined using Spearman’s rank test. Statistical significance was indicated on figures as follows: *P < 0.05, **P < 0.01, ***P < 0.001.

## Electronic supplementary material

Supplementary Dataset 1

## References

[1] Abdollahi-Roodsaz S, Abramson SB, Scher JU (2016). The metabolic role of the gut microbiota in health and rheumatic disease: mechanisms and interventions. Nature reviews. Rheumatology.

[2] Scher JU, Littman DR, Abramson SB (2016). Microbiome in Inflammatory Arthritis and Human Rheumatic Diseases. Arthritis & rheumatology (Hoboken, N.J.).

[3] McInnes IB, Schett G (2011). The pathogenesis of rheumatoid arthritis. N Engl J Med.

[4] Schett G, Coates LC, Ash ZR, Finzel S, Conaghan PG (2011). Structural damage in rheumatoid arthritis, psoriatic arthritis, and ankylosing spondylitis: traditional views, novel insights gained from TNF blockade, and concepts for the future. Arthritis research & therapy.

[5] Scher JU (2015). Decreased bacterial diversity characterizes the altered gut microbiota in patients with psoriatic arthritis, resembling dysbiosis in inflammatory bowel disease. Arthritis & rheumatology (Hoboken, N.J.).

[6] Scher JU (2013). Expansion of intestinal Prevotella copri correlates with enhanced susceptibility to arthritis. Elife.

[7] Zhang X (2015). The oral and gut microbiomes are perturbed in rheumatoid arthritis and partly normalized after treatment. Nat Med.

[8] Maeda, Y. *et al*. Dysbiosis contributes to arthritis development via activation of autoreactive T cells in the intestine. *Arthritis & rheumatology* (2016). 10.1002/art.39783 27333153

[9] Chen J (2016). An expansion of rare lineage intestinal microbes characterizes rheumatoid arthritis. Genome medicine.

[10] Honda K, Littman DR (2016). The microbiota in adaptive immune homeostasis and disease. Nature.

[11] Kamada N, Nunez G (2014). Regulation of the immune system by the resident intestinal bacteria. Gastroenterology.

[12] Lubberts E (2015). The IL-23-IL-17 axis in inflammatory arthritis. Nature reviews. Rheumatology.

[13] van den Berg WB, McInnes IB (2013). Th17 cells and IL-17 a–focus on immunopathogenesis and immunotherapeutics. Semin Arthritis Rheum.

[14] Avci AB, Feist E, Burmester GR (2016). Targeting GM-CSF in rheumatoid arthritis. Clinical and experimental rheumatology.

[15] Wicks IP, Roberts AW (2016). Targeting GM-CSF in inflammatory diseases. Nature reviews. Rheumatology.

[16] Tesmer LA, Lundy SK, Sarkar S, Fox DA (2008). Th17 cells in human disease. Immunological reviews.

[17] Mease PJ (2014). Brodalumab, an anti-IL17RA monoclonal antibody, in psoriatic arthritis. The New England journal of medicine.

[18] Mease PJ (2015). Secukinumab Inhibition of Interleukin-17A in Patients with Psoriatic Arthritis. N Engl J Med.

[19] Genovese MC (2014). A phase II randomized study of subcutaneous ixekizumab, an anti-interleukin-17 monoclonal antibody, in rheumatoid arthritis patients who were naive to biologic agents or had an inadequate response to tumor necrosis factor inhibitors. Arthritis & rheumatology (Hoboken, N.J.).

[20] Genovese MC (2014). One-year efficacy and safety results of secukinumab in patients with rheumatoid arthritis: phase II, dose-finding, double-blind, randomized, placebo-controlled study. The Journal of rheumatology.

[21] Burmester GR (2016). Association of HLA-DRB1 alleles with clinical responses to the anti-interleukin-17A monoclonal antibody secukinumab in active rheumatoid arthritis. Rheumatology (Oxford, England).

[22] Koenders MI (2005). Blocking of interleukin-17 during reactivation of experimental arthritis prevents joint inflammation and bone erosion by decreasing RANKL and interleukin-1. Am J Pathol.

[23] Sato K (2006). Th17 functions as an osteoclastogenic helper T cell subset that links T cell activation and bone destruction. J Exp Med.

[24] Kotake S (1999). IL−17 in synovial fluids from patients with rheumatoid arthritis is a potent stimulator of osteoclastogenesis. J Clin Invest.

[25] Hot A, Miossec P (2011). Effects of interleukin (IL)-17A and IL-17F in human rheumatoid arthritis synoviocytes. Annals of the rheumatic diseases.

[26] Rehaume LM (2014). ZAP-70 genotype disrupts the relationship between microbiota and host, leading to spondyloarthritis and ileitis in SKG mice. Arthritis & rheumatology (Hoboken, N.J.).

[27] Abdollahi-Roodsaz S (2008). Stimulation of TLR2 and TLR4 differentially skews the balance of T cells in a mouse model of arthritis. J Clin Invest.

[28] Wu HJ (2010). Gut-residing segmented filamentous bacteria drive autoimmune arthritis via T helper 17 cells. Immunity.

[29] Ivanov II (2009). Induction of intestinal Th17 cells by segmented filamentous bacteria. Cell.

[30] Sano T (2015). An IL-23R/IL-22 Circuit Regulates Epithelial Serum Amyloid A to Promote Local Effector Th17 Responses. Cell.

[31] Ochoa-Reparaz J (2009). Role of gut commensal microflora in the development of experimental autoimmune encephalomyelitis. Journal of immunology (Baltimore, Md.: 1950).

[32] Rosser EC (2014). Regulatory B cells are induced by gut microbiota-driven interleukin-1beta and interleukin-6 production. Nat Med.

[33] Marietta, E. V. *et al*. Human Gut-Derived Prevotella histicola Suppresses Inflammatory Arthritis in Humanized Mice. *Arthritis & rheumatology* (2016). 10.1002/art.39785 PMC5125894 27337150

[34] Hill DA (2010). Metagenomic analyses reveal antibiotic-induced temporal and spatial changes in intestinal microbiota with associated alterations in immune cell homeostasis. Mucosal Immunol.

[35] Isaac S (2017). Short- and long-term effects of oral vancomycin on the human intestinal microbiota. The Journal of antimicrobial chemotherapy.

[36] Lee YK, Menezes JS, Umesaki Y, Mazmanian SK (2011). Proinflammatory T-cell responses to gut microbiota promote experimental autoimmune encephalomyelitis. Proc Natl Acad Sci USA.

[37] Barman M (2008). Enteric salmonellosis disrupts the microbial ecology of the murine gastrointestinal tract. Infect Immun.

[38] Korganow AS (1999). From systemic T cell self-reactivity to organ-specific autoimmune disease via immunoglobulins. Immunity.

[39] Kyburz D, Corr M (2003). The KRN mouse model of inflammatory arthritis. Springer Semin Immunopathol.

[40] Maeda Y (2016). Dysbiosis Contributes to Arthritis Development via Activation of Autoreactive T Cells in the Intestine. Arthritis & rheumatology (Hoboken, N.J.).

[41] van de Stadt LA (2011). Development of the anti-citrullinated protein antibody repertoire prior to the onset of rheumatoid arthritis. Arthritis and rheumatism.

[42] Deane KD (2010). The number of elevated cytokines and chemokines in preclinical seropositive rheumatoid arthritis predicts time to diagnosis in an age-dependent manner. Arthritis and rheumatism.

[43] van der Woude D (2010). Epitope spreading of the anti-citrullinated protein antibody response occurs before disease onset and is associated with the disease course of early arthritis. Annals of the rheumatic diseases.

[44] Liu X (2016). Role of the Gut Microbiome in Modulating Arthritis Progression in Mice. Scientific reports.

[45] Ben-Amram H (2017). Tuftsin-Phosphorylcholine Maintains Normal Gut Microbiota in Collagen Induced Arthritic Mice. Frontiers in microbiology.

[46] Rogier R (2017). Aberrant intestinal microbiota due to IL-1 receptor antagonist deficiency promotes IL-17- and TLR4-dependent arthritis. Microbiome.

[47] Giongo A (2011). Toward defining the autoimmune microbiome for type 1 diabetes. The ISME journal.

[48] Gaboriau-Routhiau V (2009). The key role of segmented filamentous bacteria in the coordinated maturation of gut helper T cell responses. Immunity.

[49] Hannonen P, Mottonen T, Hakola M, Oka M (1993). Sulfasalazine in early rheumatoid arthritis. A 48-week double-blind, prospective, placebo-controlled study. Arthritis and rheumatism.

[50] Stone M, Fortin PR, Pacheco-Tena C, Inman RD (2003). Should tetracycline treatment be used more extensively for rheumatoid arthritis? Metaanalysis demonstrates clinical benefit with reduction in disease activity. The Journal of rheumatology.

[51] Morton AM (2014). Endoscopic photoconversion reveals unexpectedly broad leukocyte trafficking to and from the gut. Proc Natl Acad Sci USA.

[52] Hwang YG (2016). *Differential response of serum* amyloid A to different therapies in early rheumatoid arthritis and its potential value as a disease activity biomarker. Arthritis research & therapy.

[53] Gabay C (2016). Comparison of lipid and lipid-associated cardiovascular risk marker changes after treatment with tocilizumab or adalimumab in patients with rheumatoid arthritis. Annals of the rheumatic diseases.

[54] Migita K (2014). Effects of Janus kinase inhibitor tofacitinib on circulating serum amyloid A and interleukin-6 during treatment for rheumatoid arthritis. Clinical and experimental immunology.

[55] Shen C (2015). Increased serum amyloid A and its association with autoantibodies, acute phase reactants and disease activity in patients with rheumatoid arthritis. Molecular medicine reports.

[56] Targonska-Stepniak B, Majdan M (2014). Serum amyloid A as a marker of persistent inflammation and an indicator of cardiovascular and renal involvement in patients with rheumatoid arthritis. Mediators of inflammation.

[57] Hong C (2015). An involvement of SR-B1 mediated p38 MAPK signaling pathway in serum amyloid A-induced angiogenesis in rheumatoid arthritis. Molecular immunology.

[58] Connolly M (2016). Acute serum amyloid A is an endogenous TLR2 ligand that mediates inflammatory and angiogenic mechanisms. Annals of the rheumatic diseases.

[59] da Rocha LF (2012). Increased serum interleukin 22 in patients with rheumatoid arthritis and correlation with disease activity. The Journal of rheumatology.

[60] Ikeuchi H (2005). Expression of interleukin-22 in rheumatoid arthritis: potential role as a proinflammatory cytokine. Arthritis and rheumatism.

[61] Leipe J (2011). Interleukin 22 serum levels are associated with radiographic progression in rheumatoid arthritis. Annals of the rheumatic diseases.

[62] Benham H (2013). Th17 and Th22 cells in psoriatic arthritis and psoriasis. Arthritis research & therapy.

[63] Block KE, Zheng Z, Dent AL, Kee BL, Huang H (2016). Gut Microbiota Regulates K/BxN Autoimmune Arthritis through Follicular Helper T but Not Th17 Cells. Journal of immunology (Baltimore, Md.: 1950).

[64] Jacobs JP, Wu HJ, Benoist C, Mathis D (2009). IL-17-producing T cells can augment autoantibody-induced arthritis. Proc Natl Acad Sci USA.

[65] Shi G (2008). Phenotype switching by inflammation-inducing polarized Th17 cells, but not by Th1 cells. Journal of immunology (Baltimore, Md.: 1950).

[66] Basdeo SA (2017). Ex-Th17 (Nonclassical Th1) Cells Are Functionally Distinct from Classical Th1 and Th17 Cells and Are Not Constrained by Regulatory T Cells. Journal of immunology (Baltimore, Md.: 1950).

[67] Leipe J (2010). Role of Th17 cells in human autoimmune arthritis. Arthritis and rheumatism.

[68] Colin EM (2010). 1,25-dihydroxyvitamin D3 modulates Th17 polarization and interleukin-22 expression by memory T cells from patients with early rheumatoid arthritis. Arthritis and rheumatism.

[69] van Hamburg JP (2011). Th17 cells, but not Th1 cells, from patients with early rheumatoid arthritis are potent inducers of matrix metalloproteinases and proinflammatory cytokines upon synovial fibroblast interaction, including autocrine interleukin-17A production. Arthritis and rheumatism.

[70] van Hamburg JP (2013). IL-17/Th17 mediated synovial inflammation is IL-22 independent. Annals of the rheumatic diseases.

[71] Yamada H (2008). Th1 but not Th17 cells predominate in the joints of patients with rheumatoid arthritis. Annals of the rheumatic diseases.

[72] Miao J (2014). Frequencies of circulating IL-17-producing CD4 + CD161 + T cells and CD4 + CD161 + T cells correlate with disease activity in rheumatoid arthritis. Modern rheumatology.

[73] Chalan P (2013). Circulating CD4 + CD161 + T lymphocytes are increased in seropositive arthralgia patients but decreased in patients with newly diagnosed rheumatoid arthritis. PloS one.

[74] Jandus C (2008). Increased numbers of circulating polyfunctional Th17 memory cells in patients with seronegative spondylarthritides. Arthritis and rheumatism.

[75] Raychaudhuri SK, Saxena A, Raychaudhuri SP (2015). Role of IL-17 in the pathogenesis of psoriatic arthritis and axial spondyloarthritis. Clinical rheumatology.

[76] Joosten LA, Helsen MM, van de Loo FA, van den Berg WB (1996). Anticytokine treatment of established type II collagen-induced arthritis in DBA/1 mice. A comparative study using anti-TNF alpha, anti-IL-1 alpha/beta, and IL-1Ra. Arthritis and rheumatism.

[77] Scher JU (2016). The lung microbiota in early rheumatoid arthritis and autoimmunity. Microbiome.

[78] McDonald D (2012). An improved Greengenes taxonomy with explicit ranks for ecological and evolutionary analyses of bacteria and archaea. The ISME journal.

[79] Abdollahi-Roodsaz S (2013). Toll-like receptor 2 controls acute immune complex-driven arthritis in mice by regulating the inhibitory Fcgamma receptor IIB. Arthritis and rheumatism.

[80] Abdollahi-Roodsaz S (2007). Inhibition of Toll-like receptor 4 breaks the inflammatory loop in autoimmune destructive arthritis. Arthritis and rheumatism.

