# Supplementary material for: Alteration of the intestinal microbiome characterizes preclinical inflammatory arthritis in mice and its modulation attenuates established arthritis
Source: Sci Rep. 2017 Nov 15;7:15613. doi: 10.1038/s41598-017-15802-x (PMC5688157; doi:10.1038/s41598-017-15802-x)
Supplement: Supplementary file 1 — Supplementary Dataset 1 [file 41598_2017_15802_MOESM1_ESM.pdf]

Alteration of the intestinal microbiome characterizes preclinical inflammatory arthritis in mice and its modulation attenuates established arthritis

Rebecca Rogier[1], Heather Evans-Marin[2], Julia Manasson[2], Peter M. van der Kraan[1], Birgitte Walgreen[1], Monique Helsen[1], Liduine van den Bersselaar[1], Fons A. van de Loo[1], Peter L. van Lent[1], Steven B. Abramson[2], Wim B. van den Berg[1], Marije I. Koenders[1], Jose U. Scher[2], Shahla Abdollahi-Roodsaz[1,2,*]

[1]Department of Rheumatology, Radboud University Medical Center, Nijmegen, The Netherlands.

[2]Department of Medicine, Division of Rheumatology, New York University School of Medicine, New York, United States.

*To whom correspondence should be addressed:

Shahla Abdollahi-Roodsaz, PhD

Division of Rheumatology, NYU School of Medicine

301 East 17th Street, Room 1611A

New York, NY 10003

Experimental Rheumatology

272, Radboud University Medical Center

PO Box 9101, 6500HB, Nijmegen, The Netherlands

Tel: 212-598-6417; Fax: 212-598-7604

Email: S.Abdollahi@nyumc.org; Shahla.Abdollahi-Roodsaz@radboudumc.nl

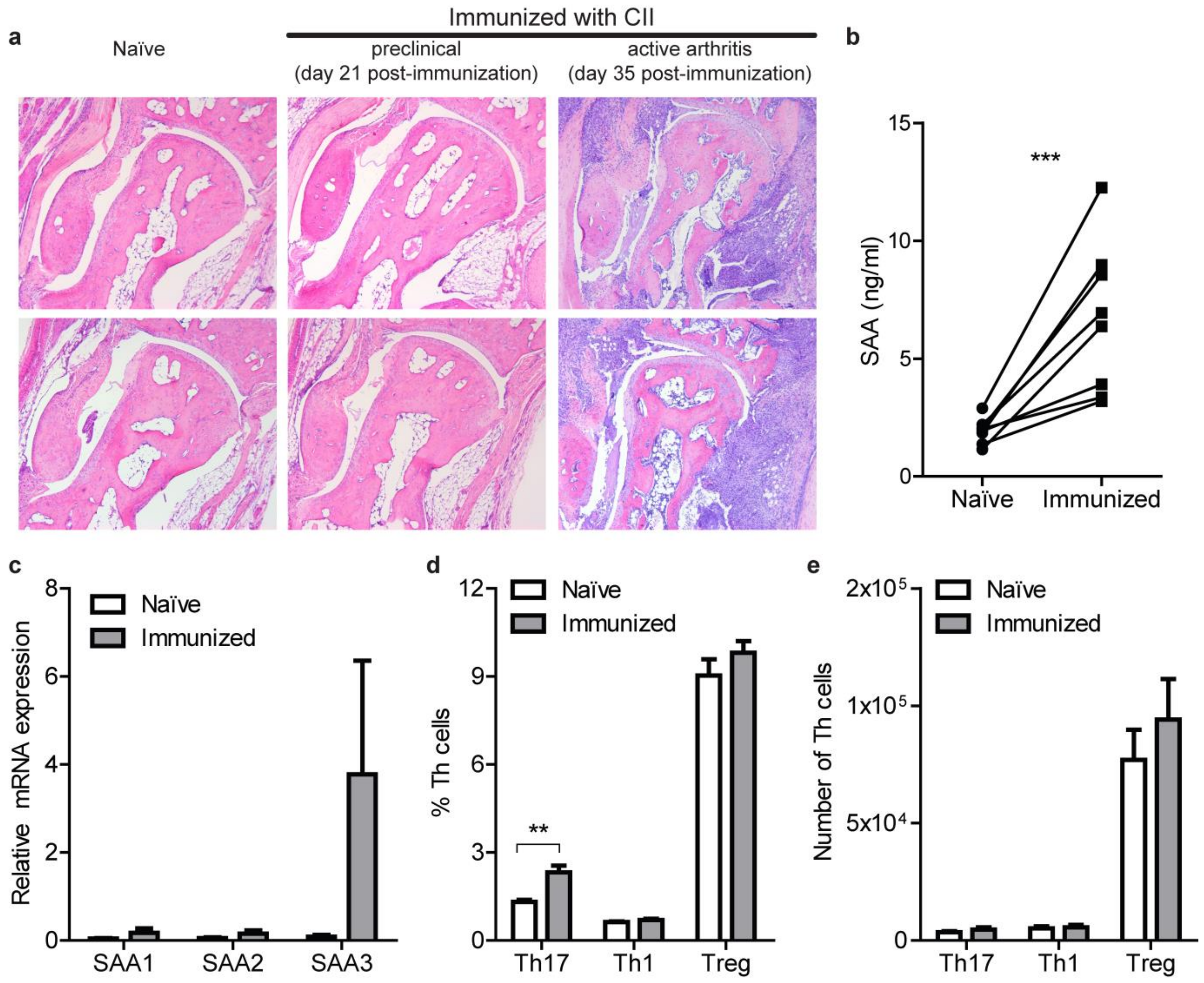

Supplementary Figure S1. Serum SAA levels significantly increased in preclinical phase of collagen induced arthritis. (A) Representative pictures of ankle joints of naïve mice and mice 21 (pre-clinical) or 35 days (active arthritis) post-immunization with collagen type II (CII). (**B**) SAA serum levels of naïve and mice 21 days after immunization (n=8). (**C**) Gene expression of SAA1, SAA2 and SAA3 in synovial biopsies of naïve (n=9) and immunized mice (n=8). Relative mRNA expression is shown as $2^{-\Delta Ct}$ *10000, corrected for GAPDH. (**D-E**) Percentage (**D**) or total number (**E**) of Th17 ($CD4^{+}TCR\beta^{+}$ IL-17^{+}) and Th1 ($CD4^{+}TCR\beta^{+}IFN\gamma^{+}$) cells isolated from popliteal lymph nodes (pLN) of naïve mice (n=6) and mice 21 days after immunization (n=6) with CII. Data are shown as mean + SEM. **p<0.01, ***p<0.001 by Mann-Whitney test.

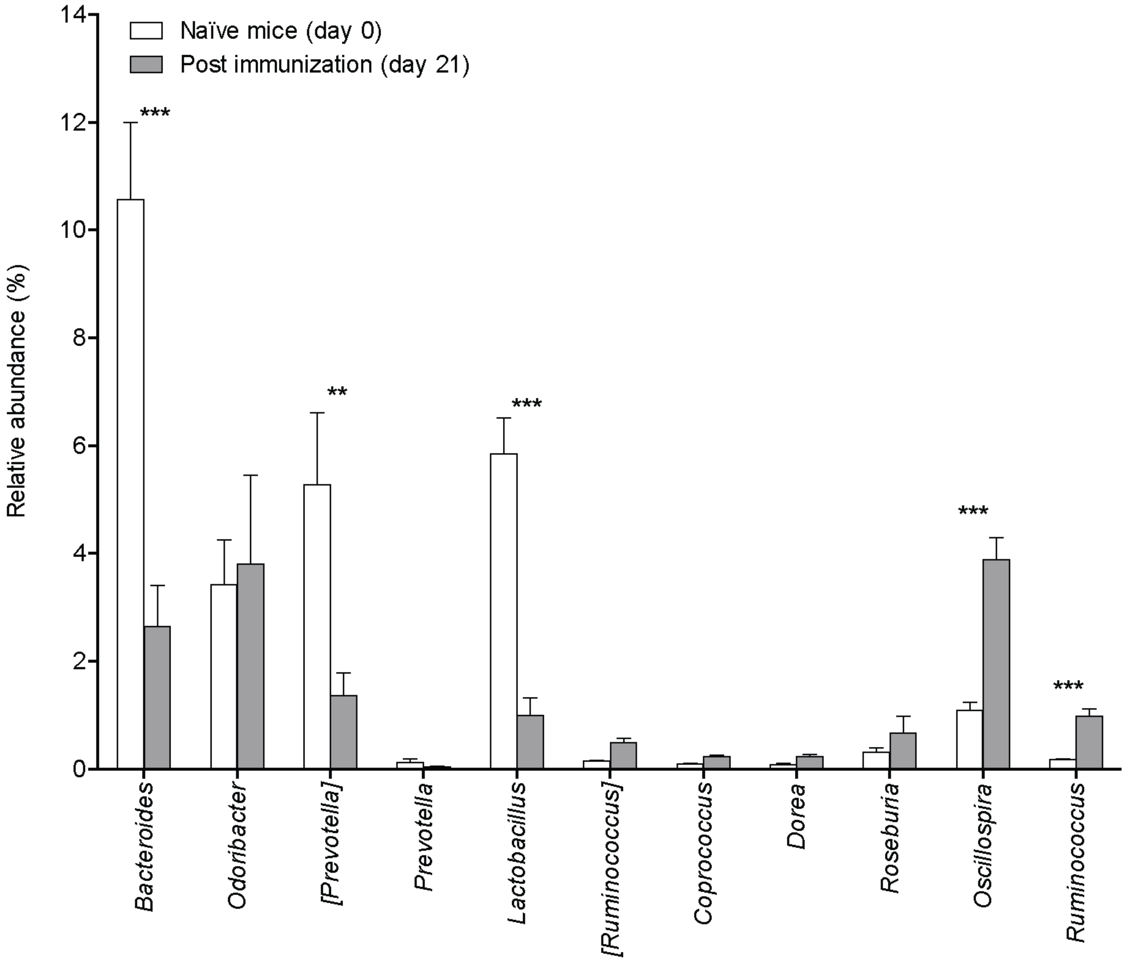

Supplementary Figure S2. Preclinical phase of collagen induced arthritis is marked by a shift in intestinal microbiota. Relative abundance of different genera present in intestinal microbiota of naïve mice (day 0) and collagen-immunized, pre-arthritic mice (day 21). Genera with a relative abundance of > 0.1% in either of the two groups are shown. Data is shown as mean +SEM of n=7 mice per group. **p<0.01, ***p<0.001, by Mann-Whitney test followed by a correction for multiple testing using the Benjamini-Hochberg procedure.

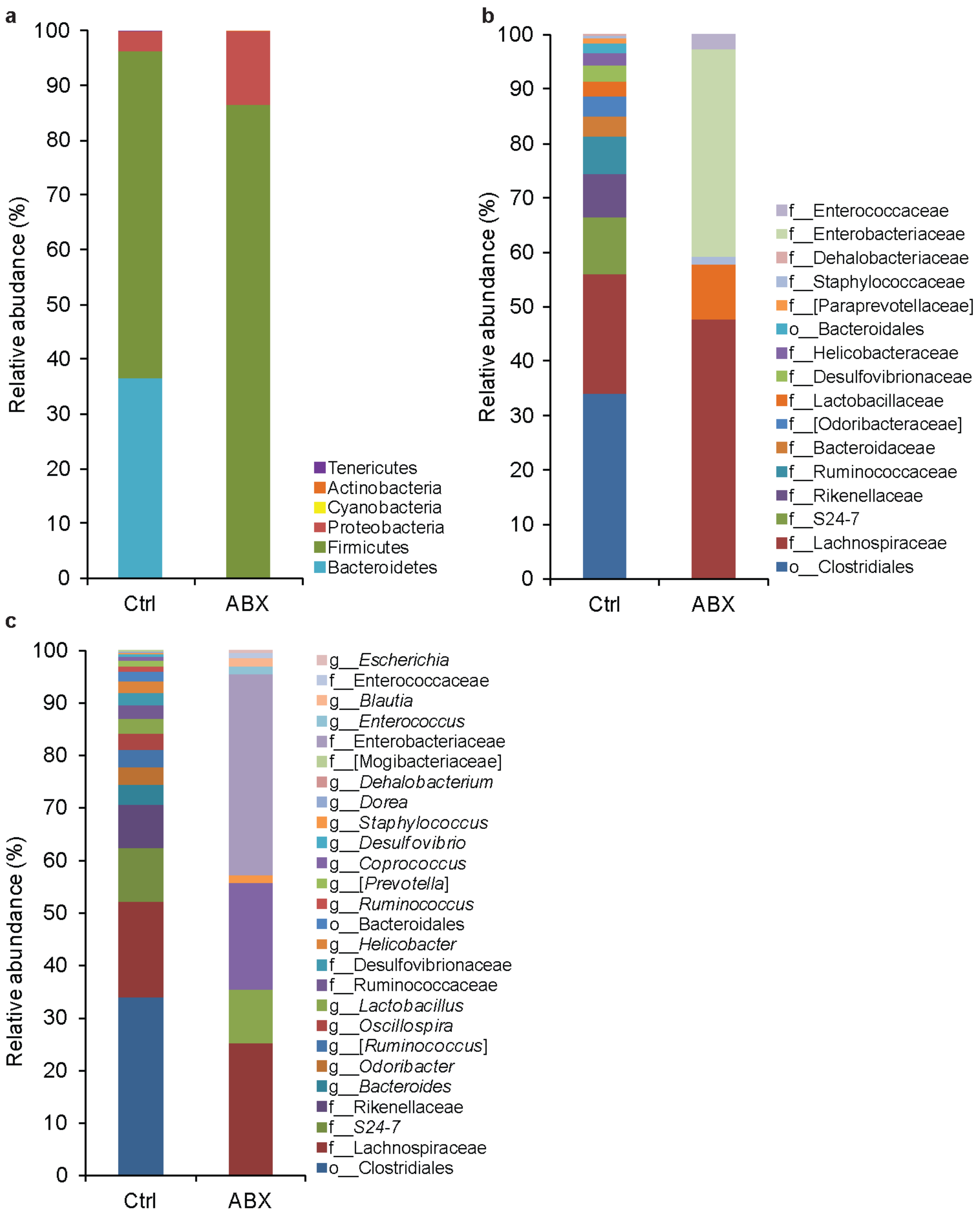

Supplementary Figure S3. Treatment with broad-spectrum antibiotics causes shift in intestinal bacterial community composition. (**A-C**) Intestinal bacterial composition at the phylum (**A**), family (**B**) and genus (**C**) level of untreated control (Ctrl, n=5) and antibiotic-treated (ABX, n=6) mice. Feces were collected 3 days after discontinuation of treatment which was the end-point of the experiment. o=order, f= family and g=genus.

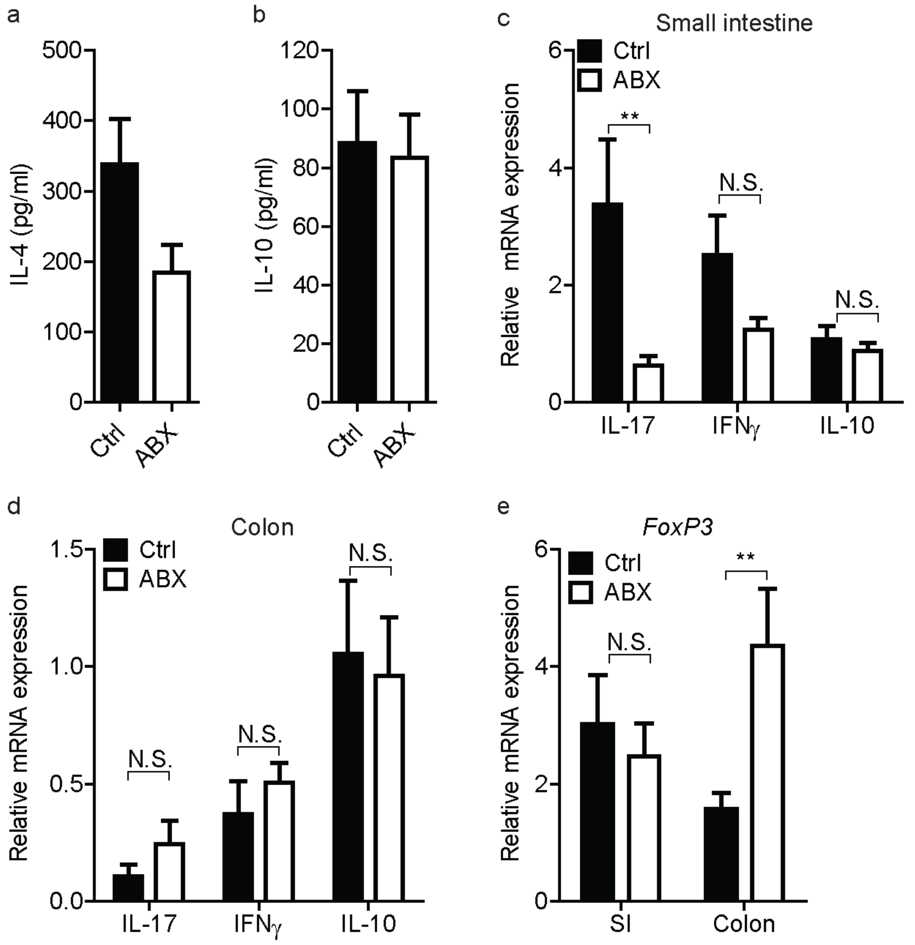

Supplementary Figure S4. Treatment with broad-spectrum antibiotics results in shift in intestinal Th cell subsets. (**A-B**) Production of IL-4 and IL-10 by lamina propria mononuclear cells of untreated control (Ctrl, n=12) and antibiotic-treated (ABX, n=10) mice with CIA upon *ex vivo* stimulation with PMA and ionomycin for 5 hours, measured by Luminex cytokine array. (**C-D**) Gene expression of IL-17, IFNγ and IL-10 in small intestine (**C**) and colon (**D**) of Ctrl (n=18) and ABX (n=19) mice, as measured by PCR. (**E**) Gene expression of FoxP3 in small intestine (SI) and colon of Ctrl and ABX mice. Relative mRNA expression is shown as $2^{-\Delta Ct}$ *10000, corrected for GAPDH. Data are shown as mean + SEM, Mann-Whitney test **p<0.01. N.S. not significant.

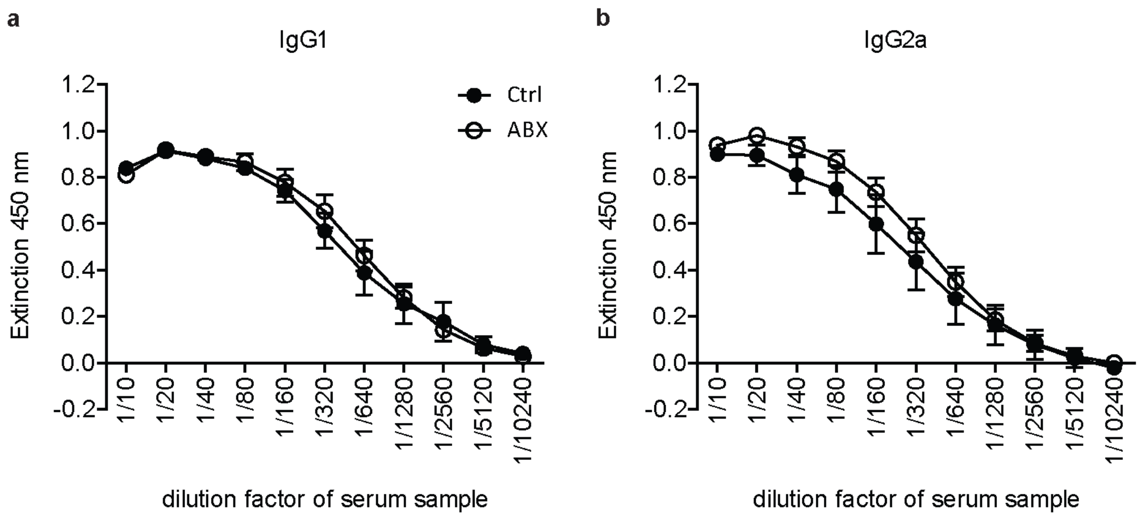

Supplementary Figure S5. Serum levels of anti-mouse collagen type II antibodies not affected by antibiotic treatment. Serum levels of IgG1 (**A**) and IgG2a (**B**) anti-type II collagen antibodies of untreated control (Ctrl) and antibiotic-treated (ABX) mice (n=7 mice per group) measured by ELISA.

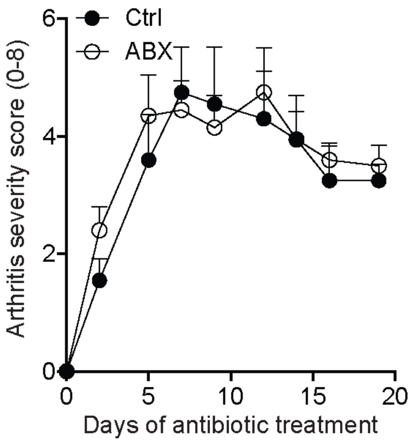

Supplementary Figure S6. Antibiotic treatment does not affect severity of serum-transfer arthritis. Macroscopic arthritis severity scores of serum-transfer arthritis (0-2 per paw) of untreated control (Ctrl) and antibiotic-treated (ABX) mice. Data is shown as mean + SEM, of n=5 mice per group.

Supplementary Table S1. Differentially abundant operational taxonomic units (OTUs) in microbiota of naïve and immunized mice. Relative abundance (percentage) of differentially present OTUs in fecal microbiota of naïve (day 0) and collagen II-immunized (day 21 post-immunization) mice. P-values were calculated using a Student's t-test followed by a correction for multiple testing using the Benjamini-Hochberg procedure; n=7 mice per group.

	Day 0		Day 21		
**Taxonomic assignment**	**Mean**	**SEM**	**Mean**	**SEM**	**P-value**
o_Bacteroidales	0.106	0.025	0.005	0.002	0.0070
o_Bacteroidales	2.342	0.656	0.149	0.042	0.0155
g_*Odoribacter*	0.207	0.062	0.046	0.030	0.0451
g_[*Prevotella*]	0.125	0.030	0.023	0.012	0.0130
g_[*Prevotella*]	4.566	1.186	1.145	0.350	0.0276
g_*Bacteroides*	0.152	0.026	0.026	0.006	0.0026
g_*Bacteroides*	0.181	0.031	0.042	0.015	0.0033
g_*Bacteroides*	1.063	0.194	0.179	0.040	0.0035
g_*Bacteroides*	0.437	0.079	0.086	0.021	0.0038
g_*Bacteroides*	0.220	0.040	0.050	0.020	0.0042
g_*Bacteroides*	2.727	0.631	0.403	0.075	0.0101
g_*Bacteroides*	0.114	0.029	0.013	0.004	0.0136
g_*Bacteroides*	0.110	0.030	0.018	0.008	0.0200
g_*Bacteroides*	3.535	0.812	1.133	0.409	0.0272
f_Rikenellaceae	0.029	0.006	0.107	0.014	0.0009
f_Rikenellaceae	0.396	0.058	0.099	0.024	0.0014
f_Rikenellaceae	0.542	0.087	0.180	0.058	0.0057
f_Rikenellaceae	1.909	0.126	0.966	0.233	0.0060
f_Rikenellaceae	0.184	0.040	0.034	0.009	0.0094
f_Rikenellaceae	0.062	0.008	0.133	0.021	0.0143
f_S24-7	0.554	0.030	0.106	0.022	0.0000
f_S24-7	0.679	0.055	0.166	0.032	0.0000
f_S24-7	1.390	0.134	0.161	0.048	0.0000
f_S24-7	0.116	0.012	0.013	0.004	0.0000
f_S24-7	0.833	0.102	0.089	0.028	0.0002
f_S24-7	1.048	0.163	0.150	0.056	0.0011
f_S24-7	0.181	0.025	0.048	0.016	0.0011
f_S24-7	3.760	0.565	0.742	0.260	0.0011
f_S24-7	0.422	0.066	0.057	0.015	0.0012
f_S24-7	0.179	0.030	0.020	0.006	0.0015
f_S24-7	0.265	0.046	0.039	0.014	0.0021
f_S24-7	3.031	0.333	1.109	0.374	0.0024
f_S24-7	0.150	0.026	0.030	0.010	0.0026
f_S24-7	0.121	0.023	0.010	0.004	0.0027
f_S24-7	0.738	0.129	0.126	0.031	0.0028
f_S24-7	2.835	0.619	0.286	0.121	0.0059
f_S24-7	0.221	0.030	0.086	0.030	0.0085
f_S24-7	0.369	0.084	0.051	0.019	0.0086

f_S24-7	0.226	0.058	0.012	0.005	0.0102
f_S24-7	0.948	0.078	0.447	0.138	0.0109
f_S24-7	0.213	0.050	0.033	0.012	0.0111
f_S24-7	0.640	0.195	0.028	0.008	0.0202
f_S24-7	0.126	0.040	0.005	0.002	0.0220
f_S24-7	0.306	0.085	0.055	0.012	0.0254
g_*Lactobacillus*	0.136	0.020	0.013	0.006	0.0005
g_*Lactobacillus*	0.290	0.061	0.077	0.024	0.0122
g_*Lactobacillus*	2.548	0.819	0.177	0.069	0.0275
g_*Lactobacillus*	1.088	0.280	0.278	0.102	0.0278
s_*reuteri*	0.100	0.013	0.029	0.012	0.0016
s_*reuteri*	0.653	0.120	0.180	0.055	0.0065
o_Clostridiales	0.364	0.104	0.969	0.089	0.0009
o_Clostridiales	0.057	0.007	0.194	0.027	0.0019
o_Clostridiales	0.013	0.004	0.109	0.026	0.0091
o_Clostridiales	0.006	0.004	0.125	0.032	0.0093
o_Clostridiales	0.128	0.012	0.279	0.042	0.0103
o_Clostridiales	0.053	0.021	0.273	0.063	0.0121
o_Clostridiales	0.189	0.114	1.033	0.275	0.0220
o_Clostridiales	0.300	0.089	0.058	0.021	0.0345
o_Clostridiales	0.040	0.010	0.227	0.071	0.0392
o_Clostridiales	0.098	0.031	0.507	0.159	0.0425
f_Lachnospiraceae	0.023	0.008	0.163	0.036	0.0075
f_Lachnospiraceae	0.104	0.030	0.287	0.048	0.0087
f_Lachnospiraceae	0.053	0.014	0.142	0.027	0.0177
f_Lachnospiraceae	0.151	0.038	0.391	0.091	0.0415
s_*gnavus*	0.078	0.015	0.157	0.020	0.0092
s_*gnavus*	0.056	0.008	0.233	0.067	0.0381
f_Ruminococcaceae	0.042	0.010	0.209	0.021	0.0001
f_Ruminococcaceae	0.035	0.010	0.192	0.026	0.0006
f_Ruminococcaceae	0.161	0.028	0.411	0.043	0.0006
f_Ruminococcaceae	0.014	0.006	0.191	0.043	0.0058
f_Ruminococcaceae	0.102	0.028	0.383	0.074	0.0082
f_Ruminococcaceae	0.153	0.032	0.831	0.208	0.0170
g_*Oscillospira*	0.040	0.012	0.238	0.032	0.0004
g_*Oscillospira*	0.096	0.014	0.369	0.044	0.0005
g_*Oscillospira*	0.043	0.013	0.181	0.039	0.0112
g_*Oscillospira*	0.091	0.035	0.511	0.125	0.0146
g_*Oscillospira*	0.027	0.006	0.108	0.024	0.0154
g_*Oscillospira*	0.068	0.016	0.175	0.042	0.0462
g_*Ruminococcus*	0.010	0.003	0.308	0.113	0.0380
f_Desulfovibrionaceae	0.062	0.008	0.293	0.019	0.0000
f_Desulfovibrionaceae	0.861	0.121	5.159	0.371	0.0000
f_Desulfovibrionaceae	0.010	0.003	0.121	0.014	0.0001
f_Desulfovibrionaceae	0.024	0.004	0.167	0.021	0.0003

Supplementary table S2. Primer sequences used for qRT-PCR.

**Gene**	**Forward**	**Reverse**
GAPDH	5′-GGCAAATTCAACGGCACA-3′	5′-GTTAGTGGGGTCTCGCTCTG-3′
IL-17A	5’-CAGGACGCGCAAACATGA-3’	5’-GCAACAGCATCAGAGACACAGAT-3’
IFNγ	5’-TCTTCTTGGATATCTGGAGGAACTG-3’	5’-AGAGATAATCTGGCTCTGCAGGAT-3’
Foxp3	5’-AGGAGAAGCTGGGAGCTATGC-3’	5’-GGTGGCTACGATTGCAGCAA-3’
SAA1	5’-TGCTGAGAAAATCAGTGATGGAA-3’	5’-GGTCAGCAATGGTGTCCTCAT-3’
SAA2	5’-GCTGACCAGGAAGCCAACA-3’	5’-GCAGTCCAGGAGGTCTGTAGTAATT-3’
SAA3	5’-GCAGCACGAGCAGGATGA-3’	5’-TCCCAGGATCAAGATGCAAAG-3’
IL-22	5’-GGTGCCTTTCCTGACCAAAC-3’	5’-CGTCACCGCTGATGTGACA-3’
UNI 16S	5’-ACTCCTACGGGAGGCAGCAGT-3’	5’-ATTACCGCGGCTGCTGGC-3’
P. copri	5’-CCGGACTCCTGCCCCTGCAA-3’	5’-GTTGCGCCAGGCACTGCGAT-3’
